# In Vitro and In Vivo Comparative Analysis of Muscle Regenerative Processes Induced by Different Microcurrent Waveforms in Skeletal Muscle Atrophy

**DOI:** 10.3390/ijms26199333

**Published:** 2025-09-24

**Authors:** Yoon-Jin Lee, Eun Sang Kwon, Yong Suk Moon, Dong Rak Kwon

**Affiliations:** 1 Department of Biochemistry, College of Medicine, Soonchunhyang University, Cheonan 31511, Republic of Korea; leeyj@sch.ac.kr; 2 Department of Medicine, College of Medicine, Keimyung University, Daegu 42601, Republic of Korea; peterkwon031218@gmail.com; 3 Department of Anatomy, Catholic University of Daegu School of Medicine, Daegu 42472, Republic of Korea; ysmoon@cu.ac.kr; 4 Department of Rehabilitation Medicine, Catholic University of Daegu School of Medicine, Daegu 42472, Republic of Korea

**Keywords:** muscle atrophy, microcurrent, skeletal muscle regeneration, waveforms, rabbit model

## Abstract

This study aimed to evaluate the regenerative effects of various microcurrent waveforms in cast-induced gastrocnemius muscle atrophy in rabbits, integrating both in vitro and in vivo analyses. After two weeks of enforced hindlimb immobilization via casting, twenty-four rabbits were divided into four groups and treated for two weeks: Group-1 (control) received sham microcurrent, Group-2 was treated with a square waveform microcurrent, Group-3 with a sine waveform, and Group-4 with a triangular waveform. Treatments were administered daily for one hour. Calf circumference, muscle thickness (via ultrasound), tibial nerve CMAP, muscle fiber CSA, and protein expression (via Western blot analysis) were assessed. Among the groups, the sine waveform microcurrent resulted in significantly enhanced recovery across all measured parameters (*p* < 0.05), showing superior improvements in muscle thickness, CMAP amplitude, and fiber CSA. Immunohistochemical analysis revealed increased expression of proliferation and angiogenesis markers, including BrdU, PCNA, VEGF, and PECAM-1, while Western blotting demonstrated robust upregulation of myogenic regulatory factors such as MyoD and myogenin. Furthermore, levels of inflammatory and apoptotic markers, including TNF-α, NF-κB, and cleaved caspase-3, and stress response proteins, including p-CHK1 and p-CHK2, were markedly reduced. Collectively, these findings indicate that sine waveform microcurrent stimulation most effectively promotes muscle regeneration in both dexamethasone-induced C2C12 myoblasts and cast-induced muscle atrophy, underscoring its therapeutic potential and warranting further studies to optimize clinical application parameters.

## 1. Introduction

Skeletal muscle plays a crucial role in human life, contributing to mobility, posture, and metabolic homeostasis. When normal function is compromised, it can lead to musculoskeletal disorders, characterized by muscle weakening, size reduction, and reduced muscle mass and fiber cross-sectional area, which can be revealed histologically, significantly reducing overall quality of life [1,2]. Moreover, these conditions contribute to increased morbidity and mortality rates, imposing substantial socioeconomic burdens on families and communities [1]. As a result, there is a strong drive to develop therapeutic approaches that can regenerate muscles and counteract muscle atrophy.

Microcurrent stimulation therapy is a treatment technique that delivers currents below 1000 μA, a level that is barely perceptible to the human body [3]. Factors influencing the muscle-regenerative effects of microcurrent include intensity, waveform, current type (direct [DC] or alternating current [AC]), treatment duration, etc. Previous studies have demonstrated that low-intensity electrical stimulation (25–500 μA) significantly increases VEGF levels and enhances muscle and skin regeneration following injury and cast-induced atrophy. Its effects were shown to be superior to those of high-intensity electrical stimulation (2.5–5.0 mA) in promoting wound and muscle healing [4,5]. Thus, these findings confirm that lower-intensity currents are more effective for regeneration.

The waveform of the electrical current has also been shown to influence muscle regeneration [6]. Among various waveforms, the sine wave (SW) was found to be more effective than the rectangular wave (RW) in rat models due to its higher conductivity in the deeper muscle regions [6]. Furthermore, SW stimulation generated significantly greater muscle strength and caused less pain compared to RW stimulation, suggesting that the sine wave may be more efficient for muscle regeneration [7].

In addition, a prior study revealed that a direct current (DC) passing through a capacitor undergoes distortion and reduction, whereas an alternating current (AC) remains unaffected under similar conditions [6]. This finding highlights the superior conductivity of an AC in deeper muscle tissues, making it more effective than a DC in stimulating muscle regeneration in rats.

Based on this evidence, we hypothesize that a sine wave microcurrent is the most effective microcurrent for muscle regeneration. While former studies have revealed the efficacy of microcurrent therapy in treating muscle atrophy in rat models [6], no research has specifically investigated the impact of different microcurrent waveforms with alternative currents on muscle regeneration in a rabbit model with cast-induced muscle atrophy. Thus, our research aims to analyze the impact of different microcurrent waveforms on regenerative processes in atrophied calf muscles of immobilized rabbits.

## 2. Results

### 2.1. Establishment of Muscle Cell Differentiation and Muscle Reduction Cell Model

**Figure 1:**
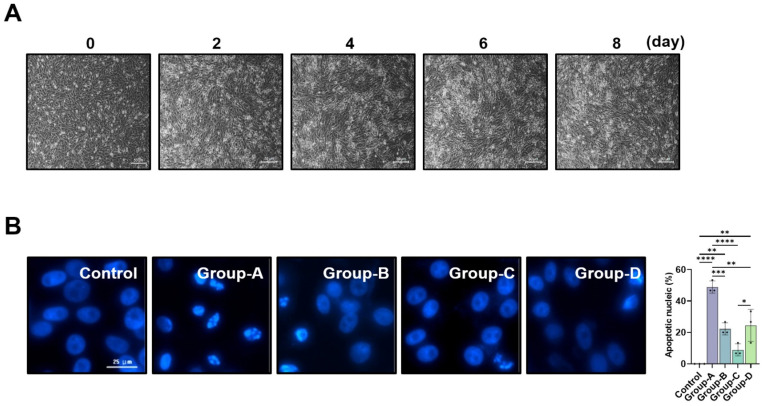
C2Cl2 myoblasts were incubated with 2% horse serum to induce myotube differentiation for different day periods (**A**), and nuclear morphology was analyzed via DAPI staining (scale bar, 25 μm) (**B**). Group-1 (control): C2C12 myoblasts with no treatment; Group-A: C2C12 myoblasts with 10 µM dexamethasone; Group-B: C2C12 myoblasts with 10 µM dexamethasone and microcurrent with square waveform for 15 min, and then left in place for 1 day; Group-C: C2C12 myoblasts with 10 µM dexamethasone and microcurrent with sine waveform for 15 min, and then left in place for 1 day; and Group-D: C2C12 myoblasts with 10 µM dexamethasone and microcurrent with triangle waveform for 15 min, and then left in place for 1 day, * *p* indicates <0.05, ** *p* indicates <0.01, *** *p* indicates <0.001, and **** *p* indicates <0.001 (post hoc testing between groups).

Before examining the effect of a microcurrent on muscle loss, C2C12 cells were differentiated from myoblasts into myotubes to establish a muscle cell differentiation and muscle loss cell model, and the morphology of the myotubes was observed. Myoblasts underwent differentiation to myotubes with 2% HS (horse serum), and the morphology of the cells was observed for up to 8 days. It was observed that differentiation into myotubes occurred from the 2nd day, and on the 6th day, complete myotube morphology was confirmed (Figure 1A).

### 2.2. DAPI Staining

Nuclear staining with DAPI revealed a rise in the number of cells with chromatin condensation and nuclear fragmentation following dexamethasone treatment, whereas microcurrent treatment reduced the occurrence of these processes (Figure 1B).

### 2.3. Clinical Parameters

There were no significant differences in mean atrophic changes (%) in the clinical, imaging, and electrophysiologic parameters between the four groups after two weeks of immobilization (*p* > 0.05, Table 1).

The mean changes (%) in the right medial and lateral GCM muscle thickness, right calf circumference, and CMAP amplitude of the right tibial nerve in Group-2, Group-3, and Group-4, indicative of regeneration, significantly exceeded those in Group-1 (*p* < 0.05, Table 2). Among these groups, Group-3 showed the greatest changes in regeneration, although the parameters between Group-3 and Group-4 did not have significant differences (*p* < 0.05, Table 2).

Motion analysis showed that total walking distance and mean walking speed were significantly greater in Group-2, Group-3, and Group-4 compared to Group-1, with no significant differences among Group-2, Group-3, and Group-4 (*p* < 0.05, Table 3).

### 2.4. Histological Examination

**Figure 2:**
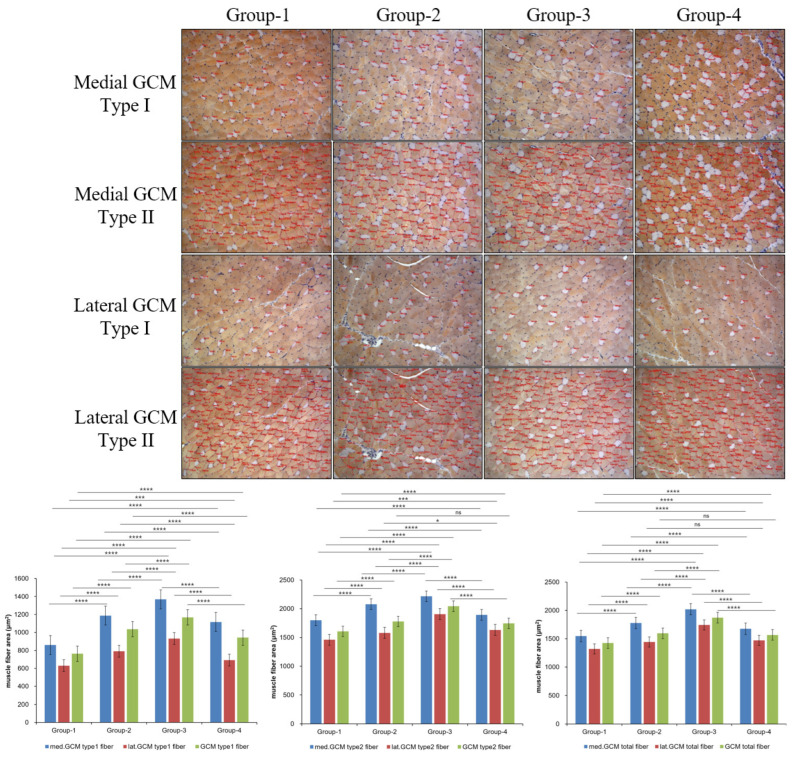
Immunohistochemical analysis of GCM (gastrocnemius muscle) fibers was conducted among the four groups, focusing on immobilized GCM muscles stained with monoclonal anti-myosin type II antibodies. The cross-sectional areas of type I and type II gastrocnemius muscle fibers, highlighted by red circles, were measured using an image morphometry program, Blue = med.GCM; Red = lat.GCM; Green = total GCM, * *p* indicates <0.05, *** *p* indicates <0.001, and **** *p* indicates <0.001 (post hoc testing between groups).

The results for the CSA of muscle fibers showed significant differences in multiple parameters. In the medial GCM, Type 1, and total muscle fibers, there were significant differences across all groups (*p* = 0.000, Figure 2), while Type 2 muscle fibers showed differences only in Groups-1 and -4 (*p* = 0.001, Figure 2). In the lateral GCM, Type 1 muscle fibers showed significant differences across all groups (*p* = 0.000, Figure 2). Type 2 muscle fibers differed significantly only in Groups-2 and -4 (*p* = 0.011, Figure 2). For all muscle fibers in the lateral GCM, all groups exhibited no significant differences (*p* = 0.000, Figure 2). For the total GCM, Type 1 muscle fibers demonstrated significant differences across all groups (*p* = 0.000, Figure 2), while both Type 2 and total muscle fibers did not exhibit significant differences in Groups-2 and -4 but did in other groups (*p* = 0.000, Figure 2).

**Figure 3:**
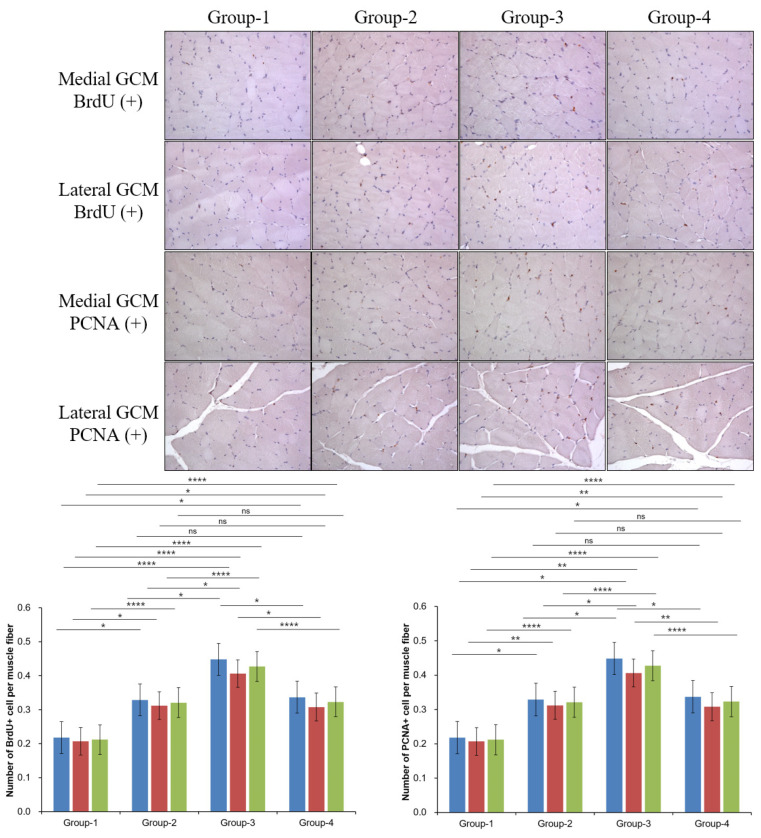
Immunohistochemical analysis of GCM (gastrocnemius muscle) fibers was conducted across the four groups, focusing specifically on immobilized GCM muscles stained with anti-BrdU and anti-PCNA antibodies. Cells or nuclei positive for BrdU and PCNA were counted along with the total number of muscle fibers within each image, Blue = med.GCM; Red = lat.GCM; Green = total GCM, * *p* indicates <0.05, ** *p* indicates <0.01, and **** *p* indicates <0.001 (post hoc testing between groups).

**Figure 4:**
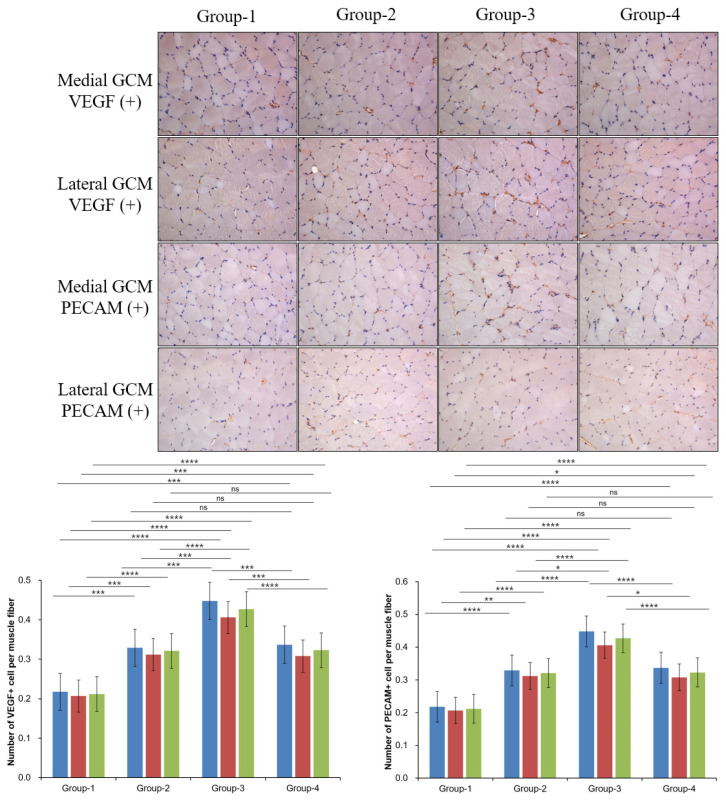
Immunohistochemical analysis of GCM (gastrocnemius muscle) fibers across the four groups involved examining immobilized GCM muscles stained with anti-VEGF and anti-PECAM-1 antibodies. The number of cells or nuclei that were positive for VEGF and PECAM-1 was counted, along with the total number of muscle fibers present in each image¸ Blue = med.GCM; Red = lat.GCM; Green = total GCM, * *p* indicates <0.05, ** *p* indicates <0.01, *** *p* indicates <0.001, and **** *p* indicates <0.001 (post hoc testing between groups).

The findings from the immunohistochemical analysis showed group-specific differences in BrdU, PCNA, PECAM, and VEGF markers. BrdU staining differed significantly in the medial and lateral GCM for Groups-1 and -3 (*p* = 0.000, Figure 3 and Figure 4), but not Groups-2 and -4. The intergroup comparative analysis also demonstrated a similar pattern (*p* = 0.05, Figure 3 and Figure 4). For the total BrdU ratio (%), Groups-2 and -4 did not demonstrate significant differences, whereas the other groups consistently did (*p* = 0.000, Figure 3). PCNA staining indicated no significant differences in Groups-2 and -4 for the medial GCM, while the other groups did (*p* = 0.05, Figure 3). In the lateral GCM, Groups-1 and -3 differed significantly (*p* = 0.000, Figure 3), while Groups-2 and -4 did not. For the total PCNA ratio (%), Groups-2 and -4 had no significant differences, whereas others did (*p* = 0.000, Figure 3).

PECAM staining showed no significant differences in Groups-2 and -4 for both medial and lateral GCM, but significant differences were observed in comparisons involving Groups-1 and -2 (*p* = 0.01, Figure 4) and Groups-1 and -4 (*p* = 0.05, Figure 4). Comparisons among other groups also demonstrated statistically significant differences (*p* = 0.000, Figure 4). VEGF staining revealed no significant differences in Groups-2 and -4 for both medial and lateral GCM. Similarly, Groups-1 and -3 did not differ significantly (*p* = 0.000, Figure 4) (*p* = 0.01, Figure 4). For the total VEGF ratio (%), Groups-2 and -4 showed no significant differences, while the other groups consistently did (*p* = 0.000, Figure 4).

### 2.5. Western Blot

#### 2.5.1. Effects of Microcurrent Waveforms on Muscle Growth, Anti-Atrophy Signaling Pathway, and Expression of Proteins Related to Inflammation In Vitro and In Vivo

**Figure 5:**
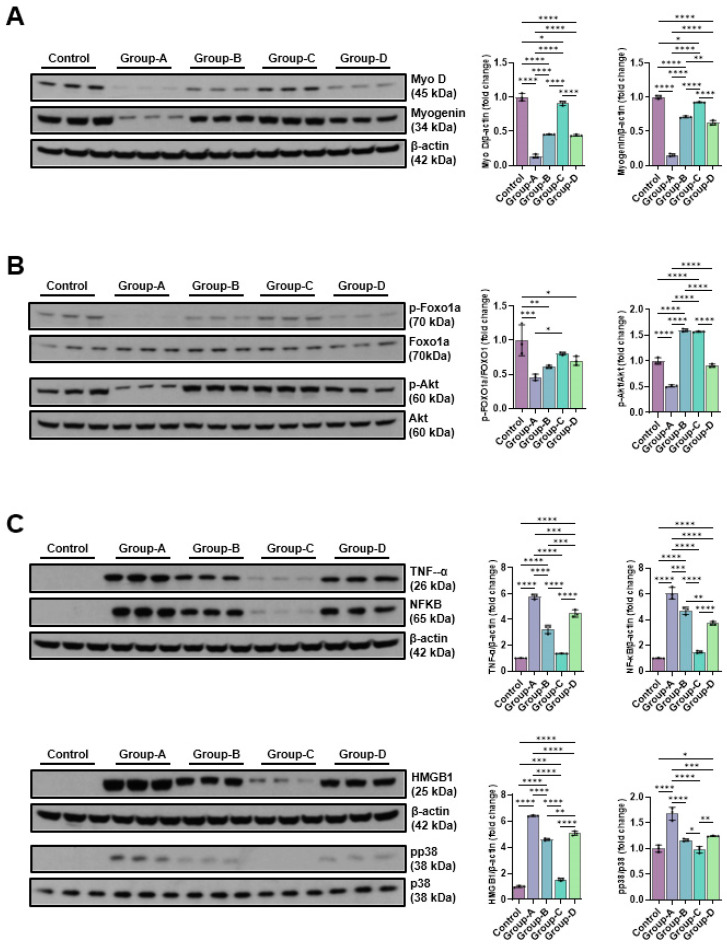
Western blotting outcomes of muscle production-related proteins (Myo D and myogenin) (**A**), muscle atrophy suppression signaling pathway-related proteins (p-Foxo1a and p-Akt) (**B**), and pro-inflammatory expression-related proteins (TNF-α, NFκB, HMGB1, and pp38) (**C**). Group-1 (control): C2C12 myoblasts with no treatment; Group-A: C2C12 myoblasts with 10 µM dexamethasone; Group-B: C2C12 myoblasts with 10 µM dexamethasone and microcurrent with square waveform for 15 min, and then left in place for 1 day; Group-C: C2C12 myoblasts with 10 µM dexamethasone and microcurrent with sine waveform for 15 min, and then left in place for 1 day; and Group-D: C2C12 myoblasts with 10 µM dexamethasone and microcurrent with triangle waveform for 15 min, and then left in place for 1 day. * *p* indicates <0.05, ** *p* < 0.01, *** *p* < 0.001, and **** *p* < 0.0001 (post hoc intergroup tests).

The change in protein expression levels for muscle cell production was confirmed through Western blotting. As a result, when the control group was considered 1, the protein expression levels of Myo-D and myogenin decreased to 0.14 ± 0.03 and 0.15 ± 0.02, respectively, when dexamethasone was applied (Group-A), and MC treatment after dexamethasone treatment was shown to increase the expression level. In particular, the sine wave treatment (Group-C) resulted in an increase similar to the control (0.91 ± 0.03 and 0.92 ± 0.01, respectively) (Figure 5A). To evaluate the mechanism of action of microcurrents on muscle atrophy inhibition, C2C12 myotubes were preprocessed with 10 μM dexamethasone for 24 h, and then treated with microcurrent for 15 min. After reacting for 24 h, proteins were collected via RIPA, and Akt and FOXO1a proteins, which are factors of the signaling pathway linked to protein synthesis and degradation, were confirmed through Western blotting. Dexamethasone treatment significantly decreased the p-Akt level, whereas microcurrent treatment increased p-Akt and p-FOXO1a; notably, treatment with the sine wave recovered levels similar to the control. p-FOAO1a was 1.00 ± 0.22 in the control group, 0.46 ± 0.04 in Group-A, 0.61 ± 0.02 in Group-B, 0.80 ± 0.02 in Group-C, and 0.70 ± 0.07 in Group-D; p-Akt was 1.00 ± 0.06 in the control group, 0.52 ± 0.02 in Group-A, 1.60 ± 0.02 in Group-B, 1.57 ± 0.01 in Group-C, and 0.91 ± 0.03 in Group-D (Figure 5B). To analyze the impact of microcurrent therapy on the expression of inflammatory proteins, the expression levels of TNF-α, NF-kB, pp38, and HMGB1 were compared using Western blotting. TNF-α was 1.00 ± 0.01 in the control group, 5.77 ± 0.19 in Group-A, 3.23 ± 0.33 in Group-B, 1.36 ± 0.01 in Group-C, and 4.47 ± 0.28 in Group-D; NF-kB was 1.00 ± 0.03 in the control group, 6.06 ± 0.46 in Group-A, 4.68 ± 0.29 in Group-B, 1.49 ± 0.09 in Group-C, and 3.71 ± 0.14 in Group-D. In addition, pp38 was 1.00 ± 0.07 in the control group, 1.68 ± 0.12 in Group-A, 1.17 ± 0.02 in Group-B, 0.98 ± 0.06 in Group-C, and 1.24 ± 0.01 in Group-D; HMGB1 was 1.00 ± 0.07 in the control group, 6.40 ± 0.05 in Group-A, 4.60 ± 0.08 in Group-B, 1.55 ± 0.10 in Group-C, and 5.06 ± 0.17 in Group-D. In Group-A, the dexamethasone treatment group, the expression levels of inflammation-related proteins significantly rose in comparison to the control group, but the expression levels decreased following microcurrent treatment; in particular, Group-C showed values similar to the control group (Figure 5C).

**Figure 6:**
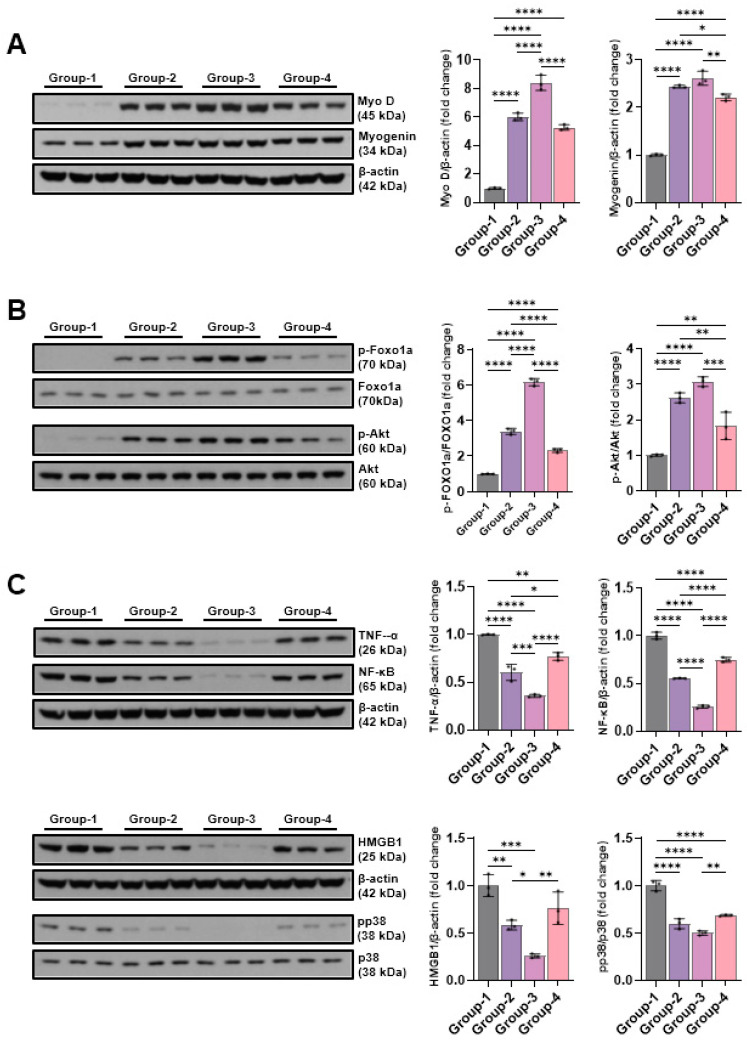
Western blot results of muscle production-related proteins ((**A**): Myo D and myogenin), muscle atrophy suppression signaling pathway proteins ((**B**): p-Foxo1a and p-Akt), and pro-inflammatory proteins ((**C**): TNF-α, NFκB, HMGB1, and pp38). Group-1 (control): IC and sham MC after CR for 2 weeks; Group-2: IC and MC after CR with square waveform for 2 weeks; Group-3: IC and MC after CR with sine waveform for 2 weeks; Group-4: IC and MC after CR with triangle waveform for 2 weeks. β-actin and p38 were applied as a loading control. * *p* indicates <0.05, ** *p* < 0.01, *** *p* for <0.001, and **** *p* < 0.0001 (intergroup post hoc tests).

The findings from the Western blot analysis revealed group-specific differences in Myo-D and myogenin. The Myo-D level was 1.00 ± 0.05 in the control group (Group-1), 6.02 ± 0.27 in Group-2, 8.40 ± 0.53 in Group-3, and 5.27 ± 0.20 in Group-4. Groups -1 and -2, -3, and -4 differed significantly, while Groups -3 and -4 did not. The myogenin level was 1.00 ± 0.02 in the control group (Group-1), 2.44 ± 0.03 in Group-2, 2.61 ± 0.15 in Group-3, and 2.21 ± 0.07 in Group-4. Groups-1 and -2, -3, and -4 differed significantly, while Groups-2 and -3 showed no significant differences (Figure 6A). Changes in the levels of p-Foxo1a and p-Akt, factors of the muscle atrophy suppression signaling pathway, were measured using Western blotting. p-Foxo1a and p-Akt levels were increased via microcurrent treatment, and, in particular, Group-3, which was treated with the sine waveform, showed the highest increases to 6.16 ± 0.21 and 3.07 ± 0.14, respectively (Figure 6B). The TNF-α, NF-κB, pp38, and HMGB1 expression levels were comparatively analyzed with Western blotting to outline the impact of different microcurrent waveforms treatment of pro-inflammatory expression. The TNF-α levels were 1.00 ± 0.00, 0.60 ± 0.08, 0.36 ± 0.01, and 0.77 ± 0.04; the NF-κB levels were 1.00 ± 0.04, 0.55 ± 0.00, 0.26 ± 0.02, and 0.75 ± 0.02 in Groups-1, -2, -3, and -4, respectively. The pp38 levels were 1.00 ± 0.05, 0.60 ± 0.05, 0.50 ± 0.02, and 0.69 ± 0.01; the HMGB1 levels were 1.00 ± 0.11, 0.58 ± 0.05, 0.26 ± 0.02, and 0.76 ± 0.17 in Groups-1, -2, -3, and -4, respectively. The expression levels of inflammation-indicative proteins decreased following microcurrent treatment, and Group-3, treated with sine waveforms, showed the most significant decrease (Figure 6C).

#### 2.5.2. Effects of Microcurrent Waveforms on Angiogenic Factors, Epithelial–Mesenchymal Transition (EMT), DNA Damage Markers, and Apoptosis Markers In Vitro and In Vivo

**Figure 7:**
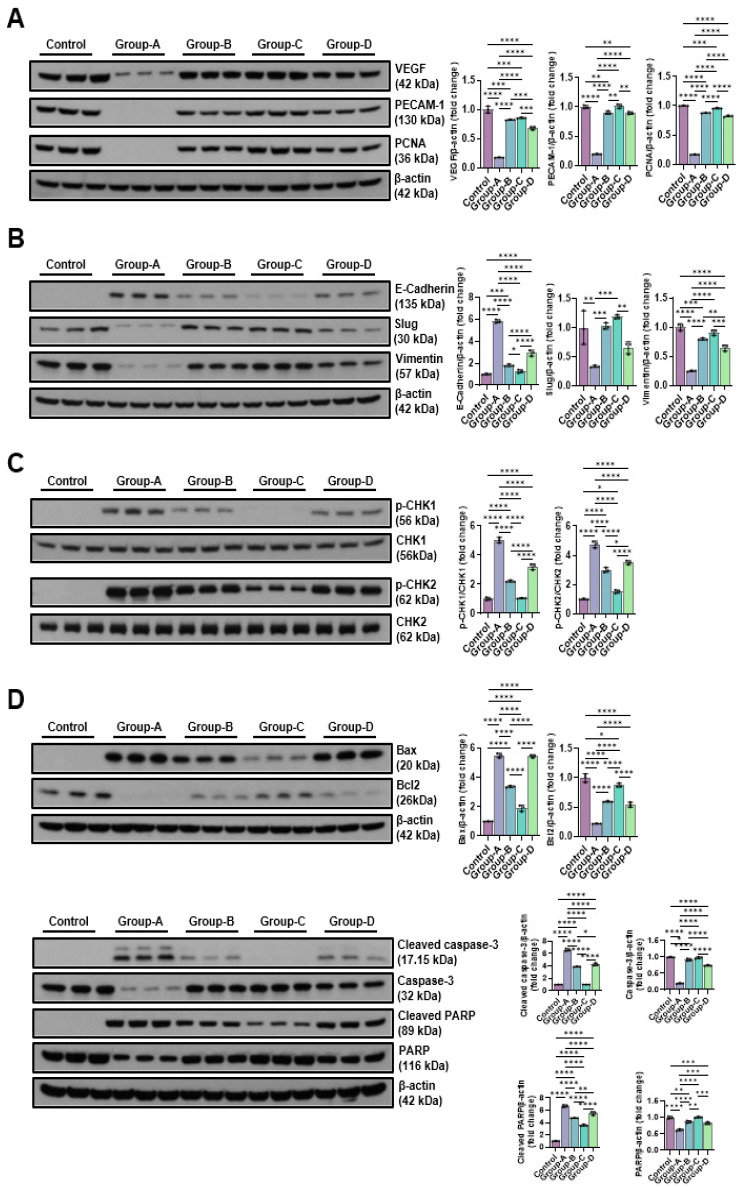
Western blot results of angiogenic factor-related proteins (VEGF, PECAM-1, and PCNA) (**A**), EMT expression-related proteins (E-cadherin, slug, and vimentin) (**B**), DNA damage-indicative proteins (p-CHK1 and p-CHK2) (**C**), and apoptosis-related proteins (BAX, Bcl-2, cleaved caspase-3, caspase-3, cleaved-PARP, and PARP) (**D**). Group-1 (control): C2C12 myoblasts with no treatment; Group-A: C2C12 myoblasts with 10 µM dexamethasone; Group-B: C2C12 myoblasts with 10 µM dexamethasone and microcurrent with square waveform for 15 min, and then left in place for 1 day; Group-C: C2C12 myoblasts with 10 µM dexamethasone and microcurrent with sine waveform for 15 min, and then left in place for 1 day; and Group-D: C2C12 myoblasts with 10 µM dexamethasone and microcurrent with triangle waveform for 15 min, and then left in place for 1 day. * *p* indicates <0.05, ** *p* < 0.01, *** *p* < 0.001, and **** *p* < 0.0001 (post hoc intergroup tests).

In our research, the expression levels of VEGF, PECAM-1, and PCNA were measured using Western blotting after microcurrent treatment in cells with dexamethasone-induced muscle atrophy. As shown in the figure, VEGF was 1.00 ± 0.06 in the control group, 0.17 ± 0.01 in Group-A, 0.82 ± 0.01 in Group-B, 0.85 ± 0.01 in Group-C, and 0.68 ± 0.03 in Group-D; PECAM-1 was 1.00 ± 0.03 in the control group, 0.19 ± 0.01 in Group-A, 0.90 ± 0.03 in Group-B, 1.01 ± 0.04 in Group-C, and 0.89 ± 0.02 in Group-D; and PCNA was 1.00 ± 0.01 in the control group, 0.16 ± 0.00 in Group-A, 0.88 ± 0.00 in Group-B, 0.96 ± 0.01 in Group-C, and 0.82 ± 0.01 in Group-D. Dexamethasone treatment significantly reduced the expression of VEGF, PECAM-1, and PCNA, but increased the expression levels that were decreased following MC treatment. In particular, the sine wave treatment group (Group-C) showed values similar to the control group (Figure 7A). To analyze the effect of microcurrents on EMT expression in cells with dexamethasone-induced muscular atrophy, the expression levels of E-cadherin, slug, and vimentin were compared using Western blotting. E-cadherin was 1.00 ± 0.06 in the control group, 5.82 ± 0.15 in Group-A, 1.81 ± 0.09 in Group-B, 1.27 ± 0.15 in Group-C, and 2.92 ± 0.28 in Group-D; slug was 1.00 ± 0.29 in the control group, 0.33 ± 0.02 in Group-A, 1.03 ± 0.05 in Group-B, 1.19 ± 0.04 in Group-C, and 0.66 ± 0.11 in Group-D; and vimentin was 1.00 ± 0.06 in the control group, 0.25 ± 0.01 in Group-A, 0.79 ± 0.02 in Group-B, 0.91 ± 0.05 in Group-C, and 0.64 ± 0.05 in Group-D. The sine wave treatment group (Group-C) restored the values that were changed following dexamethasone treatment, similar to the control group (Figure 7B). The p-Chk1 and p-Chk2 levels were assessed via Western blotting to analyze the impact of microcurrents on DNA damage. Group-A, treated with dexamethasone, demonstrated significantly higher phosphorylation of Chk1 and Chk2 (5.03 ± 0.19 and 4.74 ± 0.23, respectively) than the control, but the protein expression of p-Chk1 and p-Chk2 decreased following microcurrent treatment. Particularly, the protein expression of Group-C was hindered, similar to the control group (1.05 ± 0.03 and 1.51 ± 0.11, respectively) (Figure 7C). To analyze the impact of MC according to the type of muscle cell apoptosis in a dexamethasone-induced atrophy cell model, the expression levels of major regulatory factors related to apoptosis, such as Bax, Bcl-2, caspase-3, cleaved caspase-3, PARP, and cleaved PARP, were examined. As a result, the dexamethasone-induced atrophy group (Group-A) showed elevated expression levels of caspase-3, PARP, and Bax, while the expression of Bcl-2 decreased. Meanwhile, among the microcurrent treatments, the sine wave treatment group (Group-C) recovered similarly to the control (Figure 7D).

**Figure 8:**
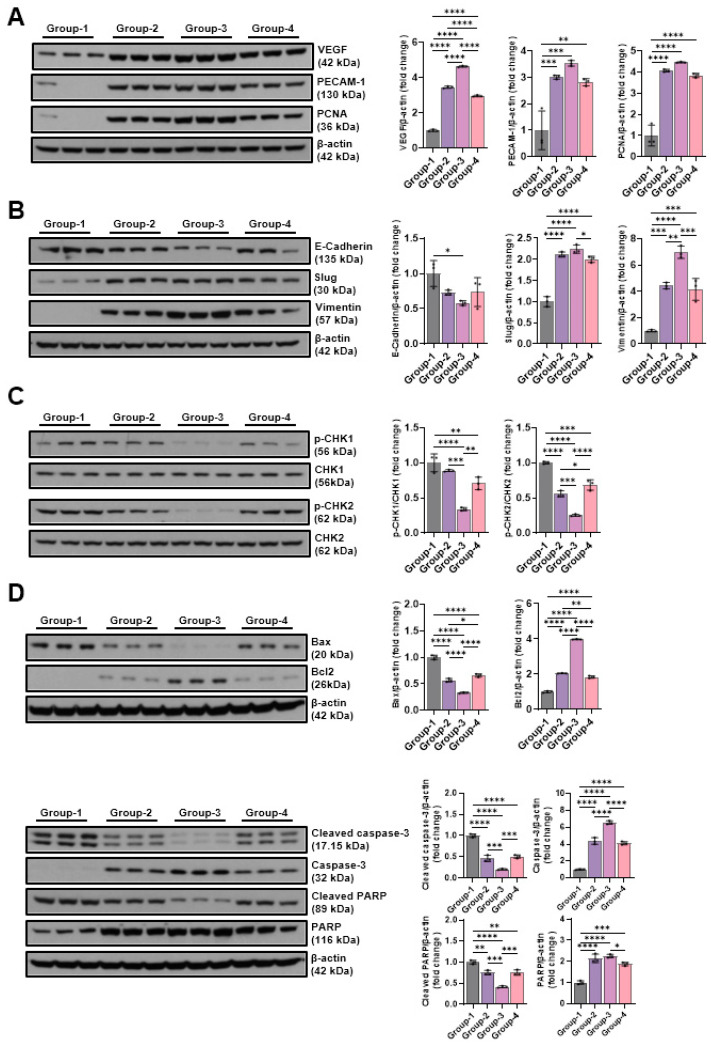
Western blotting outcomes of angiogenic factor-related proteins ((**A**): VEGF, PECAM-1, and PCNA), EMT expression-indicative proteins ((**B**): E-cadherin, slug, and vimentin), DNA damage-indicative proteins ((**C**): p-CHK1 and p-CHK2), and apoptosis-indicative proteins ((**D**): BAX, Bcl-2, cleaved caspase-3, caspase-3, cleaved-PARP, and PARP). Group-1 (control): IC and sham microcurrent (MC) after cast removal (CR) for 2 weeks; Group-2: IC and MC with square waveform after CR for 2 weeks; Group-3: IC and MC with sine waveform after CR for 2 weeks; Group-4: IC and MC with triangle waveform after CR for 2 weeks. β-actin was applied as a loading control. * *p* indicates <0.05, ** *p* < 0.01, *** *p* < 0.001, and **** *p* for < 0.0001 (post hoc tests between groups).

The expression changes in VEGF, PECAM-1, and PCNA, the most important factor in angiogenesis, were covered via Western blot analysis. VEGF expression increased to 3.46 ± 0.06 in Group-2, 4.63 ± 0.04 in Group-3, and 2.96 ± 0.05 in Group-4 compared to Group-1 (1.00 ± 0.05), and, in particular,, Group-3 treated with sine waveforms showed the highest expression. PECAM-1 and PCNA were also the highest in Group-3 at 3.54 ± 0.11 and 4.46 ± 0.03, respectively (Figure 8A). Changes in E-cadherin, slug, and vimentin were analyzed via Western blotting to investigate the effect of different microcurrent waveforms on epithelial–mesenchymal transition expression. E-cadherin expression significantly decreased to 0.73 ± 0.04 in Group-2, 0.58 ± 0.03 in Group-3, and 0.74 ± 0.21 in Group-4 compared to Group-1 (1.00 ± 0.18), and, in particular,, Group-3, which was treated with sine waveforms, showed the lowest expression. The slug levels were 1.00 ± 0.12, 2.11 ± 0.06, 2.24 ± 0.10, and 2.00 ± 0.07, and vimentin levels were 1.00 ± 0.08, 4.42 ± 0.24, 6.97 ± 0.47, and 4.14 ± 0.83 in Groups-1, -2, -3, and -4, respectively (Figure 8B). To analyze the effect of different microcurrent waveforms on DNA damage, the changes in p-CHK1 and p-CHK2 were confirmed through Western blot analysis. Microcurrent significantly reduced phosphorylation in CHK1 and CHK2, and although p-CHK1 did not differ significantly between Groups-1 and -2, p-CHK2 protein expression decreased during microcurrent treatment. Particularly, the protein expression level of Group-3 was the lowest (Figure 8C). To analyze the impact of microcurrent waveforms on apoptosis, changes in the expression rates of apoptosis-related factors, cleaved caspase-3, caspase-3, cleaved PARP, PARP, BAX, and Bcl-2, were determined via Western blotting. The level of cleaved caspase-3 decreased in Group-2 (0.46 ± 0.07), Group-3 (0.20 ± 0.01), and Group-4 (0.50 ± 0.04) compared to Group-1 (1.00 ± 0.04), and the level of cleaved PARP also decreased in Group-2 (0.75 ± 0.05), Group-3 (0.41 ± 0.02), and Group-4 (0.75 ± 0.06) compared to Group-1 (1.00 ± 0.04). In addition, Bax levels were as follows: Group-2 (0.57 ± 0.03), Group-3 (0.33 ± 0.01), and Group-4 (0.66 ± 0.03). Compared to Groups-2 and -4, Group-3, treated with a sine waveform, showed the largest decrease in the markers related to apoptosis (Figure 8D).

## 3. Discussion

The most noteworthy outcome of this investigation is the enhanced recovery effect observed in atrophied GCM muscle tissues following sine wave electrical stimulation compared to treatments using square or triangular waveforms. Among the various stimulation types applied after immobilization-induced muscle wasting, the sine waveform yielded the most favorable improvements in both structural and functional indicators of muscle regeneration. These findings were consistently demonstrated across multiple assessment modalities, including ultrasound-based muscle thickness measurements, tibial nerve compound muscle action potentials, and histological analysis of muscle fiber cross-sectional area.

Moreover, this regenerative advantage of sine wave stimulation was corroborated in an in vitro model of dexamethasone-induced atrophy using C2C12 myotubes, where sine wave-treated groups exhibited superior preservation of myotube diameter and morphology relative to other waveform conditions. These outcomes collectively suggest that sine wave stimulation can offer a more efficacious approach for mitigating muscle atrophy in both in vivo and cellular models.

In both in vitro and in vivo models of muscle atrophy, sine wave stimulation consistently produced superior outcomes compared to square and triangular waveforms. Western blot analysis of myogenic regulatory factors revealed that sine wave treatment significantly upregulated MyoD and myogenin expression, which are the key drivers of myoblast differentiation and muscle regeneration. Simultaneously, it attenuated apoptosis by decreasing levels of cleaved caspase-3, cleaved PARP, and BAX, while increasing the expression of anti-apoptotic Bcl-2, full-length caspase-3, and PARP. These outcomes indicate that sine wave stimulation can promote muscle cell survival and regeneration by modulating apoptotic pathways [8].

In addition, the expression of angiogenesis-associated proteins, including VEGF, PECAM-1, and PCNA, was markedly elevated in the sine wave group. Notably, VEGF plays a vital role in endothelial proliferation and neovascularization, while PECAM-1 contributes to capillary density and tissue repair [9,10,11]. Sine wave stimulation was also shown to enhance p-Akt and p-Foxo1a expression, suggesting its involvement in muscle-preserving signaling cascades that are known to counteract atrophy-associated pathways [12].

Immunohistochemical analysis performed in vivo further supported these findings, revealing group-specific enhancements in BrdU, PCNA, VEGF, and PECAM-1 expression in the sine wave-treated animals. These markers collectively indicate increased cellular proliferation, angiogenic activity, and tissue regeneration.

Moreover, sine wave therapy effectively suppressed pro-inflammatory signaling molecules, including TNF-α, NF-κB, HMGB1, and phosphorylated p38 MAPK. These mediators are known to exacerbate muscle degradation and impair regeneration [13,14]. Furthermore, sine wave-treated groups showed reduced expression of DNA damage-related proteins p-CHK1 and p-CHK2, implicating its protective effects on genomic integrity during recovery. Finally, EMT-related protein analysis revealed decreased expression of mesenchymal markers slug and vimentin, alongside preserved E-cadherin levels. These changes suggest that sine wave therapy may help limit fibro-adipogenic or stromal cell infiltration and reduce fibrotic transition during skeletal muscle regeneration.

Taken together, these outcomes indicate that sine wave stimulation confers a broad spectrum of therapeutic effects, including pro-regenerative, anti-apoptotic, anti-inflammatory, angiogenic, and genomic protective benefits. Its multifactorial action profile highlights sine wave as a promising modality for mitigating muscle atrophy and enhancing recovery in both cellular and animal models.

In a previous study [15], triangular, sine, and square waveforms were comparatively analyzed during the functional electrical stimulation (FES) of the upper extremities. The triangular waveform was found to require the lowest average current to produce the same movement and was associated with less discomfort compared to the square waveform. These findings suggest that the gradual increases and decreases in the current amplitude inherent to triangular waveforms may help reduce abrupt changes in stimulation, thereby minimizing patient discomfort.

In contrast, our study found the sine waveform to be the most effective. This discrepancy may be attributed to the differences in current intensity. While conventional FES typically uses stimulation intensities in the mA range to induce muscle contraction, our study used 50 µA. This substantial difference in current levels may have contributed to the differing responses observed between the triangular and sine waveforms.

Compared to the hindlimb unloading (HU) model widely used in rodents, the cast-induced immobilization model employed in this study offers distinct advantages for studying skeletal muscle atrophy. A HU model, while effective in mimicking microgravity or reduced weight-bearing conditions, does not replicate joint fixation or localized neuromuscular inactivity as seen in clinical settings [16]. In contrast, cast-induced immobilization results in more pronounced and site-specific muscle disuse, which better reflects clinical conditions, such as casting or post-operative immobilization [17,18].

Furthermore, the use of rabbits allows for higher-resolution ultrasound imaging and electrophysiological assessment due to their larger muscle mass, thereby enhancing translational relevance. These features make a cast-induced immobilization model a more physiologically and clinically representative system for evaluating anti-atrophic interventions.

Collectively, these features suggest that the rabbit cast-immobilization model provides a more clinically relevant and physiologically accurate platform for evaluating therapeutic strategies aimed at mitigating disuse-induced muscle atrophy.

No adverse outcomes were identified, which can be attributed to the fact that microcurrent delivers currents at the microampere scale, approximating the endogenous electrical levels observed in normal tissues.

In clinical practice, immobilization frequently occurs after fractures, surgical procedures, or prolonged hospitalization, leading to rapid muscle wasting and functional decline. Therefore, developing safe and effective strategies to accelerate muscle regeneration in these contexts is of high clinical importance. In the present study, microcurrent stimulation not only promoted early regenerative responses but also showed no adverse effects, likely due to its application at the microampere level, which mimics physiological bioelectric signals. This safety profile suggests that microcurrent therapy may also have preventive potential in conditions such as sarcopenia, extending its utility beyond immobilization-induced muscle atrophy.

### Study Limitations

This research has some limitations that warrant consideration. First, the total experimental period was restricted to four weeks, including baseline assessments, two weeks of immobilization, and a two-week intervention phase. As such, the long-term regenerative effects of different microcurrent electrical waveforms remain widely unclear. Extended observation periods are needed to determine whether the observed benefits persist over time. In addition, although previous studies have reported satellite cell activation only after longer remobilization phases, our follow-up was limited to two weeks, and therefore later regenerative events could not be fully addressed.

Second, only a single current intensity (50 μA) was applied in our study. This study did not examine the effects of varying microcurrent amplitudes, and it is possible that different intensities may elicit distinct physiological responses. Future research should explore a range of stimulation intensities to establish optimal parameters for muscle recovery. Our investigation primarily focused on waveform differences, and thus, the current amplitude was not systematically varied.

Third, the duration of daily stimulation was fixed at one hour, which may not represent the most effective treatment schedule. Investigating different session lengths and frequencies can provide further insight into dose-dependent therapeutic effects.

Moreover, while molecular and histological markers were analyzed, the functional evaluations were limited. Motion analysis (total walking distance and mean walking speed) was conducted, but other important measures, including muscle strength, endurance, and circulating inflammatory cytokines, were not assessed. Incorporating these parameters in future studies would enhance the translational relevance of the results.

Lastly, this study was conducted using a limited sample size and only one animal species, restricting the generalizability of the findings. Further investigations with larger cohorts and diverse animal models are needed, and ultimately, clinical studies will be required to confirm the safety and preventive potential of microcurrent stimulation in conditions such as sarcopenia.

## 4. Materials and Methods

### 4.1. In Vitro Study

#### 4.1.1. In Vitro Study Design

**Figure 9:**
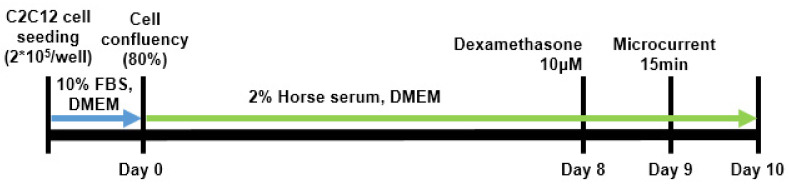
Schematic diagram of C2C12 cell culture.

C2C12 myoblasts used in the experiment were purchased from ATCC^®^ (C2C12 myoblasts, ATCC, Manassas, VA, USA) and cultivated in DMEM (Dulbecco’s Modified Eagle’s Medium, WELGENE, Daegu, Republic of Korea) enriched with 10% FBS (fetal bovine serum, WELGENE, Daegu, Republic of Korea) and 1% antibiotics (penicillin–streptomycin, WELGENE, Daegu, Republic of Korea) at 37 °C and 5% CO_2_. To promote myotube differentiation, once C2C12 myoblasts reached 80% confluency, the medium was replaced daily with DMEM containing 2% horse serum and 1% antibiotics for eight days. Meanwhile, muscle atrophy was induced using 10 µM dexamethasone (Sigma-Aldrich, St. Louis, MO, USA). After pretreatment with 10 μM dexamethasone for 24 h, cells were treated with dexamethasone alone or in combination with microcurrent for 15 min, and then left in place for 1 day (Figure 9).

Cells were treated with a microcurrent (MC) device (0.25 μA, 8 Hz; Flowmater Korea, Seoul, Republic of Korea) for 15 min using platinum electrodes in a CO_2_ incubator, then incubated for 24 h. C2C12 myoblasts were assigned to five groups (n = 3 per group), with a computerized randomization method: Group-1 (control): C2C12 myoblasts with no treatment; Group-A: C2C12 myoblasts with 10 µM dexamethasone; Group-B: C2C12 myoblasts with 10 µM dexamethasone and MC with square waveform for 1 day; Group-C: C2C12 myoblasts with 10 µM dexamethasone and MC with sine waveform for 1 day; Group-D: C2C12 myoblasts with 10 µM dexamethasone and MC with triangle waveform for 1 day. The sham MT appeared identical to the real stimulator but without an electrical current.

#### 4.1.2. DAPI Staining

Nuclear fragmentation and chromatin condensation were detected via 4′,6-diamidino-2-phenylindole (DAPI) staining. Cells (105 cells/well) were cultured in 6-well plates with dexamethasone (10 μM) in DMEM containing 2% horse serum at 37 °C for 48 h. Cells were distributed onto slides, covered with mounting medium and cover slips, and visually analyzed with a Leica EL6000 fluorescence microscope (microscope, Leica Microsistems GmbH, Wetzlar, Germany).

#### 4.1.3. Western Blot Analysis

A 0.2 × 0.2 × 0.2 cm tissue sample was treated with 180 μL of 1× radioimmunoprecipitation assay buffer (RIPA, 1× phosphate-buffered saline, 1% NP-40, 0.5% sodium deoxycholate, 0.1% SDS, 10 μg/mL phenylmethanesulfonyl fluoride, and a protease inhibitor cocktail tablet) and homogenized, and the proteins were separated. The latter were quantitatively analyzed at 40 μg with a bicinchoninic acid assay kit (Thermo Fisher Scientific Inc., Waltham, MA, USA). The quantified structures were segregated via SDS–polyacrylamide gel electrophoresis, with NuPAGE 4–12% bis-Tris gels (Invitrogen, Carlsbad, CA, USA), and then transferred to a 0.2 μm PVDF membrane (Cytiva, Marborough, MA, USA). The membrane was fixed with a casein-blocking buffer (Thermo Fisher scientific, Blocker Casein in PBS cat. no. 37528), and the rinsing solution, PBST, was obtained by mixing PBS and Tween 20. The membrane was fixed by adding 5% casein blocking buffer for two hours. After eliminating the solution, the follow primary antibodies were added: Myo D (1:500) (sc-71629, Santa Cruz Biotechnology, Dallas, TX, USA), myogenin (1:500) (sc-13137, Santa Cruz Biotechnology), HMGB1 (1:500), (ab79823, abcam, Cambridge, UK), TNF-α (1:500) (sc-52746, Santa Cruz Biotechnology), E-cadherin (1:500) (#3195, cell signaling, Danvers, MA, USA), slug (1:500) (#9585, cell signaling), vimentin (1:500) (#5741, cell signaling), p-CHK1 (1:500) (#2348, cell signaling), CHK1(1:500) (sc-8408, Santa Cruz Biotechnology), p-CHK2 (1:500) (#2197, cell signaling), CHK2(1:500) (sc-17748, Santa Cruz Biotechnology), PECAM-1 (sc-376764, Santa Cruz Biotechnology), PCNA(sc-56, Santa Cruz Biotechnology), VEGF (1:500) (sc-7269, Santa Cruz Biotechnology), NF-kB (1:500) (#8242, cell signaling), cleaved caspase-3 (1:500) (#9664, cell signaling), caspase-3 (1:500) (#14220, cell signaling), cleaved PARP (1:500) (#9541, cell signaling), PARP (1:500) (#9542, cell signaling), Bax (1:500) (#5023, cell signaling), Bcl-2 (1:500) (#2870, cell signaling), p-Foxo1a (ab47326, abcam), Foxo1a (1:500) (HPA001252), p-Akt (1:500) (#9271, cell signaling), Akt (1:500) (#9272, cell signaling), p-p38 MAPK (1:500) (#9211, cell signaling), p38 MAPK (1:500) (#8690, cell signaling), and β-actin (1:1000) (A2228, Sigma-Aldrich Corp.). They were placed in 5% casein-fixing buffer to react at 4 °C for 24 h. After eliminating the solution and rinsing with PBST, the secondary antibody was diluted in 5% casein blocking buffer to react at room temperature for 1 h. Then, the membrane was rinsed with PBST three times for 15 min each, ECL Western blotting Substrate was dispensed, and the X-ray film was developed. Densitometry was carried out on blots using ImageJ 1.0 software (National Institute of Health, Bethesda, MD, USA).

### 4.2. In Vivo Study

#### 4.2.1. Animal Grouping

The experimental protocol was approved by the Institutional Animal Care and Use Committee (IACUC) of the Catholic University of Daegu School of Medicine and was conducted in accordance with the committee’s guidelines for the care and use of laboratory animals (Approval No. DCIAFCR-240215-03-Y). Twenty-four male New Zealand White rabbits, 12 weeks of age, with a mean body weight of 3.3 kg (ranging from 2.8 to 3.6 kg), were housed individually in stainless steel cages under controlled environmental conditions (temperature: 23 ± 2 °C; humidity: 45 ± 10%) and had free access to tap water and a commercial rabbit diet. After a one-week adaptation period, their right legs were immobilized with a cast (IC) for two weeks. Then, the casts were removed along with the hair on the lower limbs with a commercial hair remover. Subsequently, the rabbits were assigned to four groups (n = 6 per group) using a computerized randomization method. Group-1 (control): IC for 2 weeks and sham microcurrent (MC) for the same time after cast removal (CR); Group-2: IC and MC with square waveform after CR for 2 weeks; Group-3: IC and MC after CR with sine waveform for 2 weeks; Group-4: IC and MC after CR with triangle waveform for 2 weeks. The visualized sham MT was identical to the real stimulator but with no electrical current. No medication was administered, and all the animals were sacrificed two weeks after the procedure.

#### 4.2.2. Immobilized-by-Cast (IC)

**Figure 10:**
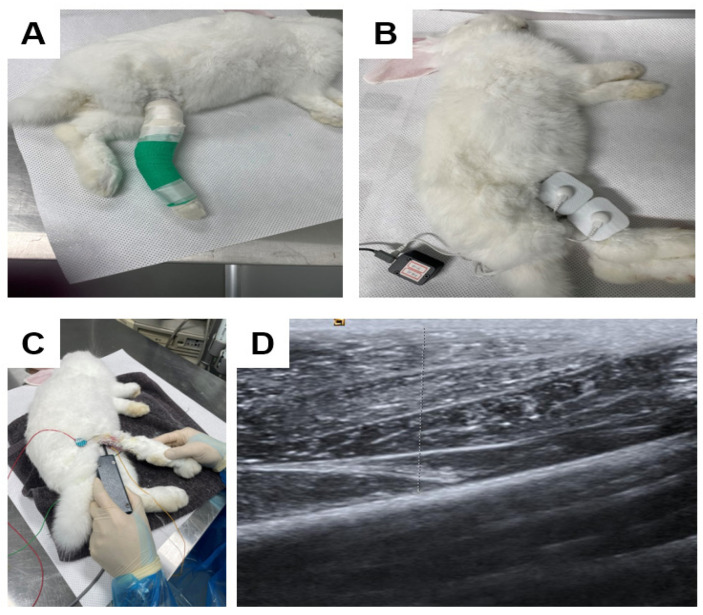
(**A**) The rabbit with the hindlimb immobilized in a cast. (**B**) The calf muscle in a cast-immobilized rabbit model. The amplitude of the CMAP in the tibial nerve. A motor nerve conduction study was carried out (**C**). The thickness of the GCM (gastrocnemius muscle) was measured using ultrasound and defined as the distance between the superficial and deep aponeuroses of the GCM muscle, indicated by up–down arrows (**D**).

For 2 weeks, the right GCM muscle underwent immobilization with a PVC plastic splint along with non-adhesive and adhesive elastic bandages (Tensoplast^®^; Smith & Nephew Medical, London, UK), following standardized IC methods. We extended right knee and ankle with a splint (Figure 10A).

#### 4.2.3. Microcurrent (MC)

The MC generator (alternating current, 50 μA, 8 Hz) was set to deliver three distinct waveform patterns, with the polarity switching every 3 s. An MC electric patch was applied to all rabbits’ skin, targeting the GCM by positioning the anode proximally and the cathode distally. Under anesthesia induced via ketamine and xylazine, microcurrent stimulation was performed to trigger the GCM of Groups-1, -2, and -3 for 60 min daily over a two-week period (Figure 10B). Notably, no visible muscle contractions were observed in the hind limbs during the microcurrent application. Throughout the experiment, the rabbits could move freely within their cages. Prior to euthanasia, the calf circumference, the complex muscle action potential (CMAP) of the tibial nerve, and the mid-belly thickness of GCM muscles were evaluated via ultrasound. The experimental protocol was adapted based on the techniques described by Moon et al. [7].

#### 4.2.4. Clinical Parameters

All the parameters were analyzed by a group-blinded physiatrist. A motor nerve conduction experiment was performed to evaluate the amplitude of the CMAP of the tibial nerve. An active electrode was positioned at the midpoint of the GCM muscle, while the reference was inserted subcutaneously at the ankle. Electrical stimulation of the nerve in the popliteal fossa was carried out, recording the greatest CMAP values after 7–10 stimuli (Figure 10C).

Calf circumference was measured with a tape, with the animals’ knee joints flexed at a 90° angle and the ankles relaxed. The thickness of the lateral and medial GCM was evaluated superficial to the deep fascia of the muscle, with real-time B-mode ultrasound (Figure 10D). Ultrasound visualization of the GCM was performed longitudinally at fixed points, comparable to the injection sites.

Alterations in CMAP amplitude, GCM thickness, and calf circumference parameters reflecting muscle regeneration were evaluated using the following formula:

(Value at 2 weeks post-treatment − Value at 2 weeks after IC)/Value at 2 weeks post-treatment ×100

The outcomes are expressed as the percentage of regenerative change on the right side.

Two weeks after MC application, we performed motion analysis; the rabbits were first released for 30 min in a 3 m^2^ open field, where they could walk for 5 min. Their motions were evaluated using a video-tracking system with a camera (Smart, Panlab, Barcelona, Spain), which recorded their activities in the horizontal plane. The total walking distance and mean walking speed over a 5 min period were assessed.

#### 4.2.5. Tissue Preparation

Following all intramuscular injections, the animals were sacrificed under general anesthesia. The GCM muscles from both sides were dissected and fixed with neutral-buffered formalin for 24 h. Subsequently, the specimens were immersed in paraffin (Paraplast; Oxford, St. Louis, MI, USA) and sectioned into 5 μm thick transversal sections for further evaluation.

#### 4.2.6. Histology and Immunohistochemistry

All histologic analyses were performed in a blinded manner, ensuring independence of evaluations, including group assignment. Muscle slices were analyzed with an Axiophot Photomicroscope (Carl Zeiss, Oberkochen, Germany). In all groups, five randomly chosen fields were acquired. Whole-muscle cross-sectional area (CSA) images of anti-myosin immune-stained muscles (at ×100 magnification) were extracted. Subsequently, we quantified anti-myosin-positive type I and type II muscle fibers using an image morphometry software program (AxioVision SE64; Carl Zeiss) and calculated their mean values.

Monoclonal anti-myosin antibodies (Skeletal, Slow; Sigma-Aldrich, St. Louis, MO, USA) were used to stain the sectioned muscles for both type I and type II fibers. Tissue sections were immunostained for proliferating cells using monoclonal anti-PCNA (Clone PC10; Santa Cruz Technologies, Dallas, TX, USA) and monoclonal anti-BrdU (Clone BU-33; Sigma-Aldrich) antibodies, and subsequently for angiogenic markers with polyclonal anti-VEGF (A-20; Santa Cruz Biotechnology, Santa Cruz, CA, USA) and anti-PECAM-1 (M-20; Santa Cruz Biotechnology) antibodies.

For BrdU staining, all the animals received 25 mg/kg of BrdU (B5002; Sigma-Aldrich) intraperitoneally and were sacrificed 24 h later, and paraffin-fixed slices were prepared.

The slices were subjected to incubation in 0.1% trypsin for 10 min at 37 °C and 1 N HCl for 30 min at 56 °C for DNA denaturation. Then, the immunohistochemical slices were rinsed with phosphate-buffered saline (PBS). Endogenous peroxidase activity was quenched by incubating the sections in 0.3% H_2_O_2_ in PBS for 30 min, followed by blocking non-specific protein binding with 10% normal horse serum in PBS (Vector Laboratories, Burlingame, CA, USA) for 30 min.

Subsequently, the slices were incubated with primary antibodies (diluted 1:100–1:500) for 2 h at room temperature and rinsed thrice with PBS. Then, the secondary antibody (biotinylated anti-mouse IgG, diluted 1:100; Vector Laboratories) was incubated in the muscle slices for 1 h at room temperature and then rinsed three times with PBS. The avidin–biotin–peroxidase complex (ABC; Vector Laboratories) was added to the slices for 1 h and then rinsed three times with PBS. Finally, the peroxidase reaction was performed using 0.05 mol/L Tris-HCl (pH 7.6) with 0.01% H_2_O_2_ and 0.05% 3,3′-diaminobenzidine (DAB; Sigma-Aldrich). The sections were subjected to counterstaining with hematoxylin and mounting. The slides were analyzed with an Axiophot Photomicroscope (Carl Zeiss, Oberkochen, Germany) and AxioCam MRc5 (Carl Zeiss).

For the assessment of BrdU, PCNA, PECAM-1, and VEGF immunostaining, images from 20 randomly selected fields per group were captured using AxioVision SE64 Rel. 4.9.1 software (Carl Zeiss). Within each image, the numbers of VEGF-, PECAM-1-, PCNA-, and BrdU-positive nuclei and the total number of muscle fibers were quantified. The expression of VEGF, PECAM-1, PCNA, and BrdU was reported as the ratio of positive cells or nuclei per 1000 muscle fibers.

#### 4.2.7. Western Blot Analysis

Calf muscle tissues were lysed in radioimmunoprecipitation assay buffer (1× PBS, 1% NP-40, 0.5% sodium deoxycholate, 0.1% SDS, 10 µg/mL phenylmethylsulfonyl fluoride, and a protease inhibitor cocktail tablet) and maintained on ice for 1 min. Homogenates were centrifuged at 10,000× *g* for 10 min at 4 °C, and the resulting supernatants were stored at −70 °C until analysis. Protein concentrations were measured using a BCA assay kit (Thermo Scientific, Waltham, MA, USA) according to the manufacturer’s instructions.

For Western blotting, 40 µg of protein from each sample was separated via SDS–polyacrylamide gel electrophoresis on NuPAGE 4–12% bis-Tris gels (Invitrogen, Waltham, MA, USA) and transferred to polyvinylidene difluoride (PVDF) membranes (GE Healthcare Life Sciences, Amersham, UK). Membranes were blocked with a casein buffer (Sigma-Aldrich, St. Louis, MO, USA) and subsequently incubated with primary antibodies. After washing with PBS containing Tween-20, membranes were incubated with HRP-conjugated anti-mouse IgG (sc-2005; Santa Cruz Biotechnology, Santa Cruz, CA, USA; dilution 1:5000). Protein bands were visualized using enhanced chemiluminescence reagents (Promega, Madison, WI, USA). β-actin (A2228; Sigma-Aldrich) was used as a loading control.

The primary antibodies used were VEGF (sc-7269; Santa Cruz Biotechnology), IL-1α (1:500, ab239517; Abcam), IL-1β (1:500, ab1832P; Merck Millipore, Burlington, MA, USA), and TNF-α (1:500, sc-52746; Santa Cruz Biotechnology). Band intensities were quantified via densitometric analysis using TINA software (Version 2.10e).

#### 4.2.8. Statistical Analysis

The initial power analysis, performed according to the pilot results, showed that the sample of 24 subjects had a statistical power of 0.8 at a significance level of 0.05. Statistical analysis was performed with the SPSS program for Windows version 22.0 (SPSS Inc., Chicago, IL, USA) and GraphPad Prism software (GraphPad Prism version 9.0, Dotmatics, Boston, MA, USA). In addition to descriptive statistics (means and standard deviations), ANOVA was applied to evaluate intra- and inter-group differences. When ANOVA indicated significant differences between the groups, Tukey’s test was performed for post hoc analysis. Mean values with 95% confidence intervals are presented, and all data are expressed as mean ± standard deviation. Statistical significance was predefined at *p* < 0.05. Post hoc power analysis indicated an estimated statistical power greater than 0.95.

## 5. Conclusions

This study demonstrates that sine waveform microcurrent stimulation exerts superior therapeutic effects on immobilization-induced skeletal muscle atrophy. Compared to square and triangular waveforms, sine wave treatment proved to be more effective in promoting muscle regeneration, reducing inflammatory signaling, suppressing apoptotic pathways, and elevating angiogenic and proliferative markers in both in vitro and in vivo models. The beneficial effects of sine wave stimulation were supported by the higher expression of myogenic proteins (MyoD and myogenin), angiogenic markers (VEGF and PECAM-1), and proliferation-related factors (PCNA and BrdU), as well as the decreased expression of pro-inflammatory cytokines and apoptotic proteins.

The obtained findings give new insights into the regenerative potential of sine waveform therapy and highlight its promise as an effective, non-invasive strategy for mitigating disuse-induced muscle loss. Future studies are warranted to optimize stimulation parameters, such as current intensity, duration, and frequency, to maximize therapeutic efficacy and facilitate clinical translation.

## Figures and Tables

**Table 1:** Atrophic changes in four groups.

Atrophic Change (%)				
Groups	Circumference on Rt Calf (cm)	CMAP on Rt. Tibial Nerve (mV)	Rt. GCM Thickness (mm)	
			Medial	Lateral
G1 (Control)	11.1 ± 4.1	37.7 ± 4.6	24.2 ± 5.7	21.1 ± 4.9
G2 (square 50 μA)	10.6 ± 2.3	35.8 ± 4.4	26.1 ± 7.2	21.5 ± 7.8
G3 (sine 50 μA)	11.0 ± 3.4	30.2 ± 7.6	26.7 ± 7.4	23.2 ± 7.4
G4 (triangle 50 μA)	11.7 ± 1.6	35.0 ± 3.6	24.1 ± 4.7	21.8 ± 7.5

Values are shown as mean ± standard deviation. There were no significant differences among the four groups. *p*-values were calculated using one-way ANOVA.

**Table 2:** Comparison of regenerative effect in four groups.

Regenerative Change (%)				
Groups	Circumference on Rt Calf (cm)	CMAP on Rt. Tibial Nerve (mV)	Rt. GCM Thickness (mm)	
			Medial	Lateral
G1 (Control)	3.8 ± 2.5 ^a^	14.7 ± 5.4 ^a^	6.8 ± 2.3 ^a^	4.5 ± 2.9 ^a^
G2 (square 50 μA)	10.9 ± 2.3 ^b^	27.8 ± 5.3 ^b^	16.9 ± 2.4 ^b^	13.4 ± 3.3 ^b^
G3 (sine 50 μA)	16.1 ± 1.6 ^c^	43.2 ± 6.4 ^c^	21.8 ± 2.7 ^c^	19.0 ± 3.0 ^c^
G4 (triangle 50 μA)	10.6 ± 1.5 ^b^	25.8 ± 7.8 ^b^	14.2 ± 3.3 ^b^	10.6 ± 3.4 ^b^

Values are demonstrated as mean ± standard deviation. a–c: Different letters on the bar stand for significantly different *p*-values < 0.05 based on Tukey’s post hoc test.

**Table 3:** Comparison of motion analysis data in four groups.

Motion Analysis		
Groups	Total Walking Distance (cm)	Mean Walking Speed (cm/sec)
G1 (control)	1275.1 ± 276.2 ^a^	4.3 ± 1.0 ^a^
G2 (square 50 μA)	3479.8 ± 531.4 ^b^	11.6 ± 1.8 ^b^
G3 (sine 50 μA)	3734.1 ± 1077.0 ^b^	12.4 ± 3.6 ^b^
G4 (triangle 50 μA)	2922.1 ± 358.9 ^b^	9.2 ± 1.7 ^b^

Values are demonstrated as mean ± standard deviation. a, b: Different letters on the bar stand for significantly different *p*-values < 0.05 based on Tukey’s post hoc test.

## Data Availability

All data generated or analyzed during this study are included in this published article.
